# Timeline and Incidence of Infectious Complications in Older Transplant Recipients During the First Year Post-Transplantation

**DOI:** 10.3390/pathogens13121061

**Published:** 2024-12-02

**Authors:** Caglayan Merve Ayaz, Serdar Ceylan, Vural Taner Yılmaz, Haydar Adanır, Özge Turhan

**Affiliations:** 1 Department of Infectious Diseases and Clinical Microbiology, Faculty of Medicine, Akdeniz University, Antalya 07070, Türkiye; merve.ayz@hotmail.com; 2 Department of Internal Medicine, Division of Geriatrics, Antalya City Hospital, Antalya 07080, Türkiye; serdarceyla@gamil.com; 3 Department of Internal Medicine, Division of Nephrology, Faculty of Medicine, Akdeniz University, Antalya 07070, Türkiye; taneryilmaz@akdeniz.edu.tr; 4 Department of Internal Medicine, Division of Gastroenterology, Faculty of Medicine, Akdeniz University, Antalya 07070, Türkiye; haydaradanir@akdeniz.edu.tr

**Keywords:** organ transplantation, aging, infections, complications, immunosuppressive treatment, graft outcomes, organ transplant rejection

## Abstract

The number of older adults undergoing organ transplantation, and waiting lists are increasing. The epidemiological data on infections in older transplant patients are scarce. The objective of the study was to investigate the incidence and distribution of infectious complications in older patients according to post-transplant periods. This retrospective study was conducted in a university hospital between 1 January 2018 and 31 March 2023. All infectious episodes were analyzed over three post-transplant periods. Forty-four patients were enrolled. The median age was 67 years (min: 65 and max: 87 years). Patients experienced a total of 98 infectious episodes. The median number of infectious events per patient was 1.0 (min: 0 and max: 8). The overall incidence rate of infectious events was 2.18 infectious episodes per 1000 transplant days. Of the patients at risk, 18.2% had 12 (12.4% of all infections) infections in the first month (9.09 episodes per 1000 transplant days), 56.8% had 52 (53.1%) infections between 1 and 6 months (7.88 episodes per 1000 transplant days), and 40.9% had 34 (35%) infections >6–12 months post-transplant (0.92 episodes per 1000 transplant days) The most prevalent type of infection was bacterial (79.6%, n = 78) followed by viral (18.4%, n = 18) and fungal (2.0%, n = 2) infections. The overall mortality rate of the 44 patients was 13.6%. The bacterial infections were more prevalent, and the incidence of infection was high during all post-transplant periods. These results may guide infection management in older transplant patients.

## 1. Introduction

Advances in surgical techniques, immunosuppression protocols, and modern antimicrobial prophylaxis have led to excellent patient and graft outcomes in solid organ transplantation (SOT) [1,2]. Long-term immunosuppressive protocols are used to prevent organ transplant rejection, thereby prolonging the life of the transplanted tissue [2]. Infectious complications are at the forefront of the conditions seen during the post-transplant period in these patients who receive intensive immunosuppressive therapy, sometimes leading to death and often requiring rapid intervention [1].

The number of older people in the world is gradually increasing, and the burden of disease is rising in parallel with increased longevity [3]. Organ function can deteriorate with increasing disease burden and aging [3]. As a result, the number of older adults undergoing transplantation and waiting lists are increasing [4]. Age is no longer an indicator, other than a numerical value of patients judged to be able to withstand the process, and transplantation is now the treatment of choice for end-stage organ disease in many institutions [4,5]. The risk of infections in transplant patients is determined by interactions between the patient’s epidemiological (e.g., countries of origin, travel, diet, habits, activities, environment, etc.) exposures and “net immunosuppression status” [6]. The source of infection may be nosocomial, technical, donor/recipient related, related to the reactivation of latent infections, relapsed, residual, opportunistic, or community-acquired, although the specific classification varies according to the post-transplantation period [6]. Post-transplantation periods in which some microorganisms frequently cause infection are summarized (Table 1) [6]. Infectious problems pose a threat to favorable outcomes for organ transplant recipients of all ages, and advanced age is one of the metabolic conditions that contribute to the status of immunosuppression and the risk of infection [4,6,7,8,9].

The extant literature data on infectious complications in older patients undergoing SOT is limited and typically reported as any patient outcome in studies. However, diagnosing and treating infections in older patients can be challenging, and the situation becomes even more complex during the transplantation process [4]. In light of the growing older patient population, it is crucial to disseminate knowledge and experience regarding transplant infections to establish a unified solution and approach. The primary objective of this study was to examine the incidence, etiology, source, agent distribution, antibiotic resistance, and mortality of infectious complications encountered in the first year following transplantation in older patients, classified according to post-transplantation periods.

## 2. Materials and Methods

### 2.1. Study Design and Settings

This retrospective, cross-sectional, cohort study was conducted in a tertiary referral university hospital in Antalya, one of Türkiye’s leading institutions for organ and tissue transplantation. Transplant patients who were 65 years of age or older undergoing kidney, liver, pancreas, and heart transplants were screened. The study was designed to follow the infectious complications of the most recently transplanted older patient for a minimum of 12 months. The majority of transplant patients received primary care at our institution after their transplant surgery. The study was approved by the Clinical Research Ethics Committee of Akdeniz University (Approval code: TBAEK-259).

### 2.2. Data Collection

**Figure 1:**
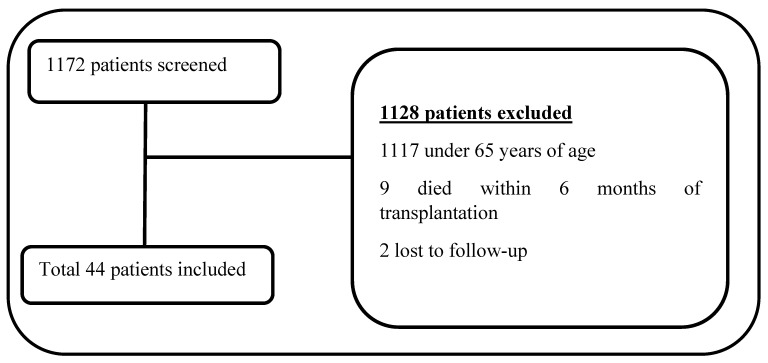
Study profile.

Medical data from patients who were followed up in inpatient and outpatient organ transplant units were examined between 1 January 2018 and 31 March 2023. Transplant patients who were 65 years of age or older at the time of surgery were included in the study. Patients who could not complete the minimum 12-month follow-up period after their transplant surgery and whose data were incomplete were excluded from the study. A total of 1172 patients were evaluated for eligibility. Of these, 55 (4.7%) patients aged ≥ 65 years were found, and 44 (3.7%) transplant patients who fulfilled the criteria were included in the study (Figure 1). For all patients, age, gender, type and date of transplantation, reason for transplantation, graft donation from deceased or living donors, the days of follow-up, co-morbidities (including diabetes mellitus, hypertension, cardiovascular diseases, rheumatologic diseases, malignancy, cerebrovascular accident, and chronic kidney, liver, and pulmonary diseases), presence of pre-transplant dialysis, model for end-stage liver disease (MELD) score for liver transplant patients, *Cytomegalovirus* (CMV) serostatus, CMV viral load (if positive), CMV treatment (if given), and number, type, and origin of the infection complications during the post-transplant period were screened.

### 2.3. Infectious Episodes and Definitions

Post-transplant infectious complications were searched retrospectively from the hospital database. All infectious episodes were analyzed during three post-transplant periods (early: 0–1 month, peak immunosuppression: 1–6 months or within 3 months of treatment of rejection, and late: >6–12 months or >3 months after treatment for a rejection), which are linked to different infectious risks in solid organ transplant recipients [2,6,10]. All patients were screened for latent or active tuberculosis, hepatitis A, B, C, and D (if necessary), cytomegalovirus, Epstein–Barr virus, varicella–zoster virus, human immunodeficiency virus, measles, rubella, mumps, syphilis, and toxoplasma. Treatment or prophylaxis was completed before transplantation, as appropriate. Repeated isolation of the same microorganism in same sample type during the ongoing infection period was not considered as a new episode. In instances where the episode of infection could not be confirmed microbiologically, clinically diagnosed infections were also included in the study. The type of infection (e.g., urinary tract infection, sinusitis, etc.) was defined in accordance with the Center for Disease Control/National Healthcare Safety Network (CDC/NHSN) surveillance definitions and criteria (11). Except for clinically diagnosed infections that did not require hospitalization, all patients were hospitalized when they had infective symptoms (such as fever, cough, purulent sputum, dysuria, chills, hypotension, and purulent drainage from incision or infectious signs and symptoms related to organs and systems), and patients who were suitable for outpatient management were discharged and followed up in outpatient clinics.

### 2.4. Microbiological Methods and Biochemical Tests

Infectious complications were studied based on their nature (bacterial, viral, or fungal), the causative agent, and the infection site. Microorganisms were detected from blood, urine, tracheal aspirate, pus, sputum, nasopharyngeal, and/or biopsy samples taken from the patients. After the samples were transferred to the appropriate transport media, they were identified by automated systems. Susceptibility and resistance profiles were studied by conventional methods. The presence of additional viral strains was confirmed through the polymerase chain reaction (PCR) methodology. In addition to culture and PCR methods, biochemical markers including procalcitonin, C-reactive protein, total blood count, and erythrocyte sedimentation rate were used to define the type of infection in all patients. At our institute, the threshold for the detection of CMV is 20 international units (IU) per milliliter. This value was employed to define a positive CMV PCR result. In SOT patients, significant clinically manifest CMV disease was defined as the presence of tissue- or organ-invasive disease or CMV syndrome, or when the CMV PCR in blood was >450 IU/mL, and antiviral treatment was initiated in the presence of these conditions [11,12]. The detection of CMV by PCR in a sample was defined as asymptomatic viral replication with no clinical, laboratory, or histological manifestations and reported only once per report period [2].

### 2.5. Immunosuppression and Prophylaxis

Immunosuppressive and preventive strategies in SOT patients are center-specific and vary according to years of experience in transplantation practice, type of transplantation, blood levels of drugs, and renal and liver function. Liver transplant patients receive mycophenolate mofetil and steroids in a tapered regimen for 12 months, trimethoprim–sulfamethoxazole, valganciclovir, and oral nystatin solution for 3 months, and cyclosporine and tacrolimus according to graft performance. In high-risk renal transplant patients, induction therapy is performed with anti-thymocyte globulin (ATG). In patients at intermediate or low risk, the choice of induction therapy is basiliximab or not. Although the regimen is updated according to the follow-up of patients, tacrolimus-based triple therapy (steroids and mycophenolic acid) is the preferred option for immunosuppressive treatment. The maintenance immunosuppressive treatment of low-risk patients is a combination of mammalian targets of rapamycin inhibitors (mTORis) and calcineurin inhibitors with steroids. In anti-microbial prophylaxis, trimethoprim–sulfamethoxazole is administered for six months, oral nystatin solution for a duration of three to six months, and valganciclovir for three months in situations where both the recipient and donor are CMV-seropositive and valganciclovir for six months in instances where the recipient is CMV-seronegative and the donor is CMV-seropositive.

### 2.6. Statistical Analysis

Nominal variables are presented as numbers and percentages, while continuous variables are presented as median, minimum (min), and maximum (max). Pearson’s χ^2^ test or Fisher’s exact test was used to compare categorical variables, and the Mann–Whitney U test was used to compare continuous numerical variables that were not normally distributed. To calculate the incidence rate of infection, we used the number of events as the numerator and the total number of days at risk for each transplant recipient during the three periods of interest: from the day of transplantation to day +30 (early period), from day +31 to day +180 or within 90 days of treatment rejection (peak immunosuppression period), and from day +181 to the end of follow-up or >91 days after treatment for the rejection period (late period) as the denominator. Infection incidence rates were calculated as the number of infections per 1000 transplant days. Statistical analysis was performed using SPSS [version 24.0 IBM SPSS Statistics, USA].

## 3. Results

In the study period, 55 consecutive patients were followed up after organ transplant surgery. Over the course of the 12-month follow-up period, 9 (16.4%) patients died, and 2 (3.6%) did not attend scheduled follow-up visits. Of the 9 patients who died, 6 (66.6%) patients died due to postoperative infectious and operation-related complications, and the remaining 3 (33.3%) patients died as a result of hyperacute/acute rejection. The total number of patients who remained in the study was 44. Twenty-six (61.0%) of these patients were female, and the median age was 67 years (min: 65 and max: 87 years). The median follow-up was 785 days (min: 372 and max: 1881 days) for kidney transplant patients and 1175 days (min: 450 and max: 2230 days) for liver transplant patients. The causes of SOT were kidney disease in 30 (68.2%) patients and liver disease in 14 (31.8%) patients. The etiological reason for transplantation is known for 11 (36.6%; 3 hypertension, 3 microscopic polyangiitis, 1, membranous glomerulonephritis, 1 interstitial nephritis, 1 gout, 1 nephrolithiasis, 1 polycystic kidney disease) kidney and 6 (42.8%; 5 chronic hepatitis B infection, 1 non-alcoholic fatty liver disease) liver transplant patients. Thirty-four (77.3%) of the patients had at least one co-morbidity other than the reason for transplantation (Table 2). Hypertension (n = 26, 59.1%), diabetes mellitus (n = 14, 31.8%), and coronary heart disease (n = 7, 15.9%) were the three most common comorbidities. No transplant patient required intensive care due to the infections. At least one episode of rejection occurred in 8 (18.2%) patients. These patients were treated with pulse steroid and/or ATG. No statistical difference was found for organ or donor type, age, gender, or the length of follow-up, and the number of comorbidities, demographics, and transplant characteristics of the patients based on post-transplant periods are summarized (Table 3).

No statistically significant differences were identified about age, gender, the duration of follow-up, the type of donor, or the number of comorbidities between patients who had undergone kidney or liver transplantation (Supplementary Table S1).

During the follow-up, the SOT recipients experienced a total of 98 infectious episodes. Of all patients, 75.0% (n = 33) experienced at least one infection, with rates of 73.3% (n = 22) for renal transplant patients and 78.6% (n = 11) for liver transplant patients. The median number of infectious events per patient was 1.0 (min: 0 and max: 8). The overall incidence rate of infectious events was 2.18 infectious episodes per 1000 transplant days. Of the patients at risk, 18.2% (n = 8) had 12 (12.4% of all infections) infections in the first month (9.09 episodes per 1000 transplant days), 56.8% (n = 25) had 52 (53.1%) infections between 1 and 6 months (7.88 episodes per 1000 transplant days), and 40.9% (n = 18) had 34 (35%) infections during >6–12 months (0.92 episodes per 1000 transplant days) following transplant surgery. Detailed information based on post-transplant infectious periods is provided (Table 4).

**Figure 2:**
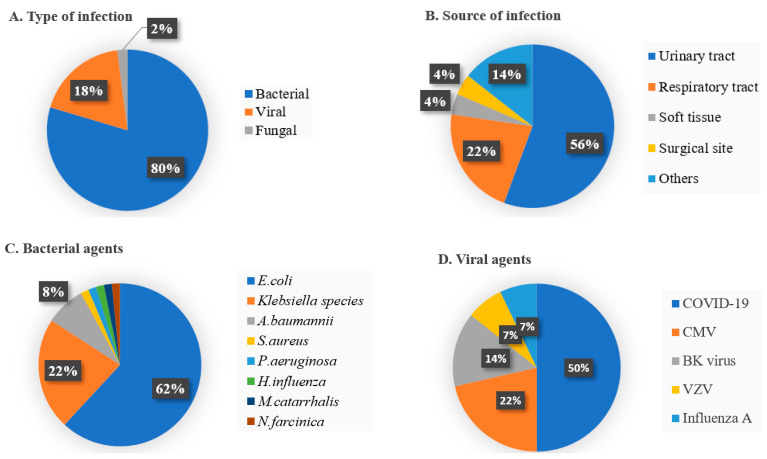
Distribution of clinically relevant infections in 44 older solid organ transplant recipients. (**A**) Cumulative incidence of type of infection, (**B**) relative percentage of source of infection, (**C**,**D**) percentage of bacterial and viral agents.

The most prevalent type of infection was bacterial, accounting for 79.6% (n = 78; 63 of them culture-proven) of all infections. A total of 18.4% (n = 18, 14 of them confirmed) of all infections were viral, including all proven viral infections. Only 2.0% (n = 2, diagnosed clinically) prevalence of fungal etiologies was observed, and no parasitic infections were found in the study population. In all periods, urinary tract infections (n = 54, 55.1%) were the most frequent infections followed by respiratory tract infections (n = 21, 21.4%), soft tissue infections (n = 4, 4.1%), and surgical site infections (n = 4, 4.1%; Figure 2). Signs and symptoms in all patients were related to the organ and system involved.

The responsible agent was identified in 78.6% (n = 77) of infectious episodes in transplant patients. Of these, 63 (81.8%) were bacterial infections and the remaining 14 (18.2%) were viral infections. COVID-19 (n = 7, 50.0%) was the most commonly detected viral etiology. All transplant patients exhibited seropositivity for CMV immunoglobulin G (Ig G) before transplantation. CMV replication was observed in 12 patients (27.3%, all of them renal transplant patients except one) during the peak immunosuppression period, only three cases of clinically significant CMV reactivation (CMV PCR > 450 IU/mL) being treated with antiviral (valganciclovir) therapy. On the other hand, a high proportion of Gram-negative organisms (n = 61, 80.2%) were isolated in identified bacterial infections. The most common bacterial etiology was *Escherichia coli* (n = 39, 63.9%), followed by *Klebsiella* species (n = 14, 22.9%), *Acinetobacter baumannii* (n = 5, 8.2%), and one case each of *Staphylococcus aureus*, *Pseudomonas aeruginosa, Haemophilus influenzae, Moraxella catarrhalis*, and *Nocardia farcinica* (Figure 2). All infections were monomicrobial, with the exception of one patient. The antibiotic susceptibilities of the identified bacteria were analyzed. *Extended spectrum beta-lactamase* (*ESBL*) production in *E. coli* isolates and *Klebsiella* species was 61.5% (n = 24) and 78.6% (n = 11), respectively. Carbapenem resistance was found in all *A. baumannii* isolates, but no colistin resistance was detected. The carbapenem resistance rate was 7.7% (n = 3) in *E. coli*, and no resistance was found in *Klebsiella* species. Methicillin resistance was not observed in *S. aureus* isolate. 

The overall mortality rate of 55 transplant patients was 29.1%, while in the study group (44 patients), it was 13.6% (6 patients; 5 in the infected group vs 1 in the uninfected group, *p* = 1.00).

## 4. Discussion

The risk of post-transplant infections remains a significant source of morbidity and mortality due to the lifelong use of immunosuppressive drugs to prevent rejection, despite numerous medical advances [7,13]. Given the paucity of knowledge regarding infectious complications in older SOT patients, we felt it was important to contribute to this field. The data presented are of significant importance, not only in providing explanations at the clinical, microbiological, and epidemiological levels but also in understanding the burden of infection in this population compared to different age groups of transplant recipients. Compared to previous studies, the main findings of this study were high infection rates [2,7,14,15]; high numbers of infectious events per patient [2]; high overall incidence of infectious episodes [2]; and high early [16], 1- and 6-month [2,16] and late infection incidence [2,16,17]. Although the incidence of infectious episodes decreased dramatically after the first month (from 9.09 episodes per 1000 transplant days to 0.92 episodes per 1000 transplant days in the late period), it was still higher. It has been observed in previous studies that patients aged ≥65 years are proportionally less represented in the transplant population. This is thought to be due to lower life expectancy [4,18,19], duration of donor organ utilization in the post-transplant period [3,18,19], the increased number of comorbid diseases and medication burden associated with older age [4,8,18], increased incidence of infection [14,15,17], and increased risk of infection-related mortality [13]. The results of this study also suggest that older transplant patients have a higher burden of infectious complications [15].

The lack of a significant difference between the presence of infection and the demographic, clinical, and transplant characteristics of the patients according to the post-transplant periods may be due to the limited number of patients included in the study cohort. 

The immune system is the primary defense against infections. The concepts of immunosenescence and inflamm-aging that occur in older adults affect the innate and adaptive immune systems, leading to increased susceptibility to infections [4,20]. It is known that persistent inflammation caused by CMV increases with aging. This is associated with poor outcomes including increased susceptibility to infections, graft loss, and mortality. In addition, the presence of comorbidities (e.g., cardiovascular disease, diabetes mellitus) is known to contribute negatively to this increased process [21,22]. Transplantation adds a new and more complex dimension to this situation, with immunosuppressive drugs given to prevent organ rejections [23]. The older age, comorbidities, and CMV-seropositive status in this study patients may be the reasons for the high infection rates. Since control of these factors is not possible, a balance between the degree of immunosuppression and the risk of infection must be maintained.

Bacterial infections are common in SOT patients, and when they are isolated in culture media, clinicians can apply targeted treatments based on the antimicrobial susceptibility results. The antibiotic susceptibility of microorganisms was also investigated, and the production of *ESBLs* and resistance to carbapenems, particularly in *Enterobacterales* and *A. baumannii*, was at an alarming level when compared the other studies [2,14,24,25,26,27,28,29,30,31]. Resistance in numerous bacteria is not specific to the older patients in the present study but is a nationwide phenomenon and has been reported by the World Health Organization and the European Centre for Disease Prevention and Control [32]. For this reason, we felt it was more appropriate to compare the resistance rates in older patients with other studies conducted in the same region to obtain an accurate perspective. In a study of 41 transplant patients with *A. baumannii* infection between 2011 and 2017, multidrug-resistant and extensively drug-resistant rates were 58.5% and 41.5%, respectively [33]. While the rate of *ESBLs* for *E. coli* in urinary tract infections was 23.5% [34], for *E. coli* and *K. pneumoniae* in bloodstream [35] and early infections [36] were 57.7%, 60.7%, 48.5%, and 32%, respectively. In another study, cefepime resistance was reported in 48.1% of *E. coli* bacteremia and 72.9% of *K. pneumoniae* [37]. The antimicrobial resistance observed in previous studies was relatively low in comparison to this study.

Similarly, to other studies, Gram-negative microorganisms predominated in the etiology of infections [2,14,16,17]. While bloodstream infections were less common [2,13,14], urinary tract infections and respiratory tract infections [16,17] were the most common infections, probably related to the type of transplanted organ (e.g., kidney transplants predominate), the degree of immunosuppression, and aging. In contrast to other studies, the prevalence of bacterial infections was reported as higher in our older transplant patients’ cohort compared with other types of infection [2,13,14].

It is well-established that there is a strong correlation between age and mortality. Age-related comorbidities, an altered immunological response and increased susceptibility to infections contribute to adverse outcomes, particularly mortality [2,3,4,5,6,7,8,9]. In comparison to other studies, our study revealed a higher overall mortality rate (16.4%) and mortality rate (13.6%) among patients who were followed up for 12 months after transplantation [7,13]. These high mortality rates may have been caused by advanced age, the COVID-19 outbreak, and being in an area with a high-resistance setting.

There are potential limitations to our study. An attempt was made to identify infectious complications in all inpatients and outpatients; it is possible that some infections were not reported and are therefore underestimated. In our sample, 68.2% had received a kidney transplant, which may influence the frequency and type of infectious complications. Our study was not designed to assess the risk of infection in the presence or absence of antimicrobial prophylaxis or to investigate possible associations between treatment preferences, outcomes, and infections. Frailty status (calculated using frailty scales or other assessment methods of physical function), which is used as an indicator of poor outcomes rather than chronological age, was not used in this study. Since the study period also included the COVID-19 outbreak period, this may have affected infection rates. Finally, because the study was cross-sectional, retrospective, and single-center and included a limited number of patients, the results cannot be generalized to other institutions and regions with different microbial epidemiology and prophylaxis strategies.

## 5. Conclusions

As life expectancy increases, organ transplantation is becoming an option suitable for older people who can tolerate the procedure. Infectious complications are common due to the lifelong use of immunosuppressive drugs. As a result of both aging and immunosuppression, infectious complications in older patients are challenging to manage. The results of the study may contribute to the management and choice of treatment based on epidemiological data of the older transplant patient population, which is expected to increase soon, by showing that bacterial infections are more common and that the incidence of infection is high in all periods after transplantation. However, multicenter prospective studies with larger numbers of patients are also needed.

## Figures and Tables

**Table 1:** The association of various microorganisms as infectious agents within post-transplant periods.

Microorganisms	Post-Transplant Period
**Virus**	
Herpes group (CMV, EBV, HHV6, 7, 8, HSV, VZV)	1–12 months and >12 months
Hepatitis viruses (HAV, HBV, HCV, HEV)	1–12 months and >12 months
Respiratory viruses (RSV, influenza, parainfluenza, etc.)	1–12 months and >12 months
Polyomavirus, papillomavirus	1–12 months and >12 months
**Bacteria**	
*Mycobacteria* (Tuberculosis and nontuberculous)	1–12 months and >12 months
* Nocardia* spp.	1–12 months and >12 months
Methicillin-resistant staphylococci	<1 month and 1–12 months
Antimicrobial-resistant enterococci	<1 month and 1–12 months
Multidrug-resistant Gram-negative bacilli	<1 month and 1–12 months
Clostridium difficile	<1 month and 1–12 months
*Listeria monocytogenes*, *Salmonella* spp., *Campylobacter* spp.	1–12 months and >12 months
Atypical respiratory pathogen (*Legionella* spp., *Mycoplasma* spp., *Chlamydia pneumoniae*)	1–12 months and >12 months
**Fungus**	
* Candida* spp.	<1 month and 1–12 months
*Aspergillus* spp.	1–12 months and >12 months
Geographic fungi (*Histoplasma capsulatum*, *Coccidioides immitis*, *Blastomyces dermatitidis*, *Paracoccidioides* species)	1–12 months and >12 months
Opportunistic molds (*Scedosporium*, agents of *Mucormycosis*, *Phaeohyphomycoses*)	1–12 months and >12 months
*Cryptococcus* spp., *Pneumocystis jiroveci*	1–12 months and >12 months
**Parasites**	
* Toxoplasma gondii*	1–12 months and >12 months
Intestinal parasites	1–12 months and >12 months
* Leishmania* spp.	1–12 months and >12 months
Others	1–12 months and >12 months

**Abbreviations:** CMV: cytomegalovirus, EBV: Epstein–Barr virus, HHV: human herpes virus, HSV: herpes simplex virus, VZV: varicella zoster virus, HAV: hepatitis A virus, HBV: hepatitis B virus, HCV: hepatitis C virus, HEV: hepatitis E virus, RSV: respiratory syncytial virus, Others: *Trypanosoma cruzi*, *Naegleria* spp.

**Table 2:** Baseline characteristics of the study population.

	All Patients (n = 44, 100%)	Uninfected Patients (n = 11, 25.0%)	Infected Patients (n = 33, 75.0%)	*p*
**Median age at transplant, y (min–max)**	67.0 (65.0–87.0)	66.5 (65.0–87.0)	67.0 (65.0–72.0)	0.91
**Female gender**	26 (59.1)	7 (58.3)	19 (59.4)	1.0
**Number of co-morbidities**				
1	17 (38.6)	4 (36.5)	13 (39.4)	1.0
2	12 (27.3)	5 (45.5)	7 (21.2)	0.26
3 or more	5 (11.4)	-	5 (15.2)	0.30
**Transplanted organ**				0.72
Kidney	30 (68.2)	9 (75.0)	21 (65.6)	
Liver	14 (31.8)	3 (25.0)	11 (34.4)	
**Type of donor**				
**Renal**				0.06
Living	28 (93.3)	6 (75.0)	22 (100)	
Deceased	2 (6.7)	2 (25.0)	-	
**Liver**				1.0
Living	13 (92.9)	3 (100)	10 (90.9)	
Deceased	1 (7.1)	-	1 100)	
**Median follow-up, d (min–max)**	907.5 (372.0–2230.0)	592.0 (385.0–1881.0)	935.0 (372.0–2230.0)	0.12
**Median MELD score for liver transplantation (min–max)**	10.5 (6.0–18.0)	13.0 (6.0–15.0)	10.0 (8.0–18.0)	1.0
**Presence of pre-transplant dialysis**	15 (34.1)	4 (33.3)	11 (34.4)	0.62
**Mortality**	6 (13.6)	1 (9.1)	5 (15.2)	1.0

**Abbreviations:** n: number, y: year, min: minimum, max: maximum, MELD: models for end-stage liver disease.

**Table 3:** Patient and transplant characteristics based on post-transplant periods.

	0–1 month			1–6 months			>6 months			Total		
Presence of Infection	No (n = 36, 81.8%)	Yes (n = 8, 18.2%)	*p*	No (n = 19, 43.2%)	Yes (n = 25 52.9%)	*p*	No (n = 26, 59.1%)	Yes (n = 18, 40.9%)	*p*	No (n = 12, 27.3%)	Yes (n = 32, 72.7%)	*p*
**Median age, y (min–max)**	67.0 (65.0–87.0)	65.5 (65.0–71.0)	0.21	66.0 (65.0–87.0)	67.0 (65.0–71.0)	0.45	67.0 (65.0–87.0)	66.0 (65.0–72.0)	0.48	66.5 (65.0–87.0)	67.0 (65.0–72.0)	0.91
**Median follow-up, d (min–max)**	897.0 (385.0–2230.0)	1471.0 (372.0–1824.0)	0.27	803.0 (385.0–1881.0)	920.0 (372.0–2230.0)	0.50	804.0 (385.0–2034.0)	955.0 (372.0–2230.0)	0.40	592.0 (385.0–1881.0)	935.0 (372.0–2230.0)	0.12
**Transplanted organ**			0.70			0.08			0.26			0.72
Kidney	25 (69.4)	5 (62.5)		17 (81.0)	13 (56.5)		16 (61.5)	14 (77.8)		9 (75.0)	21 (65.6)	
Liver	11 (30.6)	3 (37.5)		4 (19.0)	10 (43.5)		10 (38.5)	4 (22.2)		3 (25.0)	11 (34.4)	
**Type of donor**			0.46			0.57			0.26			0.18
Living	34 (94.4)	7 (87.5)		17 (89.5)	24 (96.0)		23 (88.5)	18 (100)		10 (83.3)	31 (96.9)	
Deceased	2 (5.6)	1 (12.5)		2 (10.5)	1 (4.0)		3 (11.5)	-		2 (16.7)	1 (3.1)	
**Female gender**	20 (55.6)	6 (75.0)	0.44	10 (52.6)	16 (64.0)	0.54	15 (57.7)	11 (61.1)	0.82	7 (58.3)	19 (59.4)	1.0
**Number of co-morbidities**												
1	13 (36.1)	4 (50.0)	0.69	8 (42.1)	9 (36.0)	0.76	10 (38.5)	7 (38.9)	1.0	4 (36.5)	13 (39.4)	1.0
2	11 (30.6)	1 (12.5)	0.41	6 (31.6)	6 (24.0)	0.76	8 (30.8)	4 (22.2)	0.73	5 (45.5)	7 (21.2)	0.14
3 or more	3 (8.3)	2 (25.0)	0.22	2 (10.5)	3 (12.0)	1.0	2 (7.7)	3 (16.7)	0.39	-	5 (15.2)	0.31
**Rejection episode**										1 (9.1)	7 (21.2)	0.66

**Abbreviations:** n: number, y: year, min: minimum, max: maximum, d: day.

**Table 4:** Number of infectious events after solid organ transplantation.

Infections	Total Number, (%)
Number of infectious episodes	98 (100)
0–1 month	12 (12.2)
Bacterial infections	11 (91.7)
Viral infections	1 (8.3)
1–6 months	52 (53.1)
Bacterial infections	41 (78.8)
Viral infections	11 (21.2)
>6 months	34 (34.7)
Bacterial infections	29 (85.3)
Viral infections	3 (8.8)
Fungal infections	2 (5.9)
Number of infectious episodes per patient, median (min–max)	1.0 (0–8.0)
0–1 month	0 (0–4.0)
1–6 months	1.0 (0–6.0)
>6 months	0 (0–4.0)
Patients with ≥1 infectious episode(s)	
0–1 month	8 (18.2)
1–6 months	25 (56.8)
>6 months	18 (40.9)

**Abbreviations:** min: minimum, max: maximum.

## Data Availability

The datasets used and/or analyzed during the current study is available from the corresponding author on reasonable request.

## Supplementary Materials

The following supporting information can be downloaded at: https://www.mdpi.com/article/10.3390/pathogens13121061/s1, Table S1: Baseline characteristics of liver and kidney transplant patients.

## Abbreviations

SOT	Solid organ transplantation
MELD	Model for end-stage liver disease
CMV	Cytomegalovirus
CDC/NHSN	Center for Disease Control/National Healthcare Safety Network
IU	International units
PCR	Polymerase chain reaction
ATG	Anti-thymocyte globulin
mTORi’s	Mammalian target of rapamycin inhibitors
Min	Minimum
Max	Maximum
Ig	Immunoglobulin
ESBLs	Extended spectrum beta-lactamases
COVID-19	Coronavirus disease 2019
