# Supplementary material for: Timeline and Incidence of Infectious Complications in Older Transplant Recipients During the First Year Post-Transplantation
Source: Pathogens. 2024 Dec 2;13(12):1061. doi: 10.3390/pathogens13121061 (PMC11728761; doi:10.3390/pathogens13121061)
Supplement: Supplementary file 1 [file pathogens-13-01061-s001.zip › pathogens-3322610-supplementary.pdf]

Supplementary Table S1 Baseline characteristics of liver and kidney transplant patients.

		Transplanted organ		
	**All patients (n=44, 100%)**	**Kidney (n=30, 68.2%)**	**Liver (n=14, 31.8%)**	***p***
**Median age at transplant, y (min-max)**	**67.0 (65.0-87.0)**	67.0 (65.0-87.0)	66.5 (65.0-72.0)	0.78
**Female gender**	26 (59.1)	18 (60.0)	8 (57.1)	0.86
**Number of co-morbidities**				
1	17 (38.6)	12 (40.0)	5 (35.7)	0.79
2	12 (27.3)	7 (23.3)	5 (35.7)	0.48
3 or more	5 (11.4)	5 (16.7)	0	0.16
**Infection**	33 (75.0)	22 (73.3)	11 (78.6)	1.0
**Type of donor**				1.0
Living	41 (93.2)	28 (93.3)	13 (92.9)	
Deceased	3 (6.8)	2 (6.7)	1 (7.1)	
**Median follow-up, d (min-max)**	907.5 (372.0-2230.0)	785.5 (372.0-1881.0)	1175.0 (450.0-2230.0)	0.07
**Mortality**	6 (13.6)	3 (10.0)	3 (21.4)	0.36

Abbreviations: n: number, y: year, min: minimum, max: maximum, d: day
